# Placenta-Derived Mesenchymal Stem Cells Reduce the Interleukin-5 Level Experimentally in Children with Asthma

**DOI:** 10.7150/ijms.33590

**Published:** 2019-09-20

**Authors:** Sheng-Chieh Lin, Yih-Mei Liou, Thai-Yen Ling, Ya-Hui Chuang, Bor-Luen Chiang

**Affiliations:** 1 Department of Pediatrics, Shuang Ho Hospital, Taipei Medical University, Taipei, Taiwan.; 2 Department of Pediatrics, School of Medicine, College of Medicine, Taipei Medical University, Taipei, Taiwan.; 3 Graduate Institute of Clinical Medicine, College of Medicine, National Taiwan University, Taipei, Taiwan.; 4 Department of Clinical Laboratory Sciences and Medical Biotechnology, College of Medicine, National Taiwan University, Taipei, Taiwan.; 5 Department of Pharmacology, College of Medicine, National Taiwan University, Taipei, Taiwan.; 6 Department of Laboratory Medicine, National Taiwan University Hospital, College of Medicine, National Taiwan University, Taipei, Taiwan.

**Keywords:** asthma, allergy, mesenchymal stem cell, IL-5, CD4, CD8.

## Abstract

**Background:** Mesenchymal stem cells (MSCs) have been investigated as a new treatment option for various diseases in recent years. However, the role of placenta-derived MSCs in children with asthma remains unclear. We assessed the effect of placenta-derived MSCs on T cell immune responses and cytokine IL-5 levels according to cultures in children with and without asthma.

**Study design:** We enrolled children with and without asthma and recorded asthma symptom scores in the asthma group. Blood samples from children were collected to isolate peripheral blood mononuclear cells (PBMCs) and determine the total IgE level. The PBMCs were cultured *in vitro* with or without MSCs after stimulation with human anti-CD3 and anti-CD28 antibodies (0.5 μg/mL) to evaluate the effect of placenta-derived MSCs. Flow cytometry was performed to detect the activation and proliferation of CD4+ and CD8+ T cells. Pre- and post-culture IL-5 levels were measured in all samples.

**Results:** The percentages of activation and proliferation among CD4+ and CD8+ T cells after coculture with MSCs were significantly lower in the asthma group (*P* < 0.05). IL-5 levels differed significantly between the PBMC culture and PBMC + MSC (P+S) coculture in the asthma group (*P* < 0.05). IL-5 levels differed significantly between the PBMC culture and P+S coculture in both the lower (*P* < 0.05) and higher (*P* < 0.0005) IgE asthma subgroups. IL-5 levels were also decreased in children with all severities of asthma (*P* < 0.05).

**Conclusions:** Placenta-derived MSCs exerted an anti-IL-5 effect and reduced the IL-5 level in culture in different subgroups of children with asthma.

## Introduction

Asthma is a common respiratory tract disease that manifests as an allergic and inflammatory process in children. Although several factors related to asthma are understood, its precise cause remains unknown. The complex relationships among genetic, environmental, pharmacological, and immunological factors in this disease still require further investigation 1. Asthma is relatively common, and the pathophysiology of asthma is characterized by injury, inflammation, and eventually the remodeling of airways 2. For a long time, asthma was regarded as a chronic type 2 T helper (Th2) cell-driven disease with eosinophilic airway inflammation 3. Eosinophils develop from hematopoietic CD34+ progenitor cells, and interleukin (IL)-5 is a key cytokine involved in the differentiation, proliferation, and survival of eosinophils 4. IL-5 is mostly secreted by allergen-reactive T cells, mast cells, and eosinophils 5. A study reported that CD34+/IL-5+ cells could be found in patients with asthma 4. Notably, IL-5 plays a crucial role in asthma. In recent years, stem cell transplantation has been evaluated as a disease therapy. Hematopoietic stem cell transplantation is a life-saving treatment for severe combined immunodeficiency even when an HLA-identical donor is not available 6. Tan et al. 7 reported that placenta-derived mesenchymal stem cells (MSCs) and methylprednisolone exerted a significant effect on the recovery of neurological function with neuroprotective effects in an animal model. MSCs can undergo proliferation and multiple differentiations, possess strong potential for tissue regeneration, and exert anti-inflammatory and immunomodulatory effects 2,8,9,10,11. MSCs in bone marrow can differentiate into cells that constitute multiple nonhematopoietic organs 8. Many studies have examined the use of stem cell therapy in various diseases such as acute lung injury 12, acute pancreatitis 13, and hepatic failure 14. In allergic rhinitis in adults, bone marrow-derived MSCs and induced pluripotent stem cell-derived MSCs (iPSC-MSCs) significantly suppress Th2 cells, promote regulatory T (Treg) cell responses, and inhibit lymphocyte proliferation in peripheral blood mononuclear cells (PBMCs) 15. However, Desai et al. 16 reported that MSCs presented allergens to CD4+ T cells, thus causing an increase in the production of inflammatory cytokines and the proliferation of lymphocytes in allergic rhinitis in adults. Nevertheless, no study has focused on the therapeutic effects of MSCs on asthma in children. Intravenous MSCs transplantation in murine asthma models improved the pathological features of asthma, some of which include decreased collagen deposition and inflammatory infiltration around the airway 9,17,18,19, blunt airway hyper-responsiveness 19,20,21, improved pulmonary histological scores 17,22,23, and remodeling prevention 24,25,26. MSCs and serelaxin exerted a synergistic effect to prevent airway fibrosis in an experimental model of chronic allergic airway disease 27. In a cat model of asthma, MSC-treated animals exhibited decreased airway inflammation with significantly lower bronchial wall thickening scores and lung attenuation on computed tomography images 8 months after the study 24. MSC-treated asthmatic cats also exhibited significantly decreased airway inflammation, parenchymal changes, airway hyper-responsiveness, and airway remodeling 25. Repeated administration of allogeneic adipose-derived MSCs exerted a delayed effect in preventing airway remodeling in asthmatic cats 25.

Conventionally, glucocorticoids and bronchodilators are used for the treatment of asthma. However, some children whose asthma cannot be easily controlled with the use of glucocorticoids may require alternative treatment. Promising therapeutic methods that showed favorable outcomes in animal models of asthma should be converted into clinical practice in humans. However, the exact mechanism of function of placenta-derived MSCs in children remains unclear and requires further evaluation. This study evaluated the effect of placenta-derived MSCs on the activation and proliferation of CD4+ T cells and CD8+ T cells in children with and without asthma. In addition, we evaluated the mechanisms through which MSCs exerted an anti-IL-5 effect and attempted to understand the potential of MSC therapy in children with asthma.

## Materials and Methods

### Participants and data collection

In this study, we enrolled children who had been diagnosed with asthma and children without asthma. Inclusion criteria for the asthma group were based on the Global Initiative for Asthma guidelines 26. Children who had acute asthma exacerbation and did not use steroids within 1 week before the study were included in the asthma group. Children without asthma were included in the nonasthma group. Children who had parasitic infections or hepatic disorders were excluded. This study was approved by the Institutional Review Board of Taipei Medical University and was conducted in accordance with the Declaration of Helsinki. After obtaining parental written informed consent, we collected a 5-mL sample of peripheral blood and recorded children's age and sex. We enrolled 68 children (28 girls and 40 boys), 45 and 23 of whom were included in the asthma and nonasthma groups, respectively. The sex ratio was 0.67:1 (18 girls and 27 boys) and 0.77:1 (10 girls and 13 boys) in the asthma and nonasthma groups, respectively. At the time of the study, all children were between the ages of 2 and 12 years, with a mean age of 6 years (6.00 ± 0.26). The average ages of children in the asthma and nonasthma groups were 5.88 years (5.88 ± 0.32) and 6.25 years (6.25 ± 0.44), respectively. No statistically significant intergroup age difference was observed (*P* = 0.50). The demographics of the children are listed in Table 1.

### Asthma symptom score

The asthma symptom score was determined for children with asthma. The score ranged from 0 to 16 points for the following symptoms. Nighttime cough: 0 = absent, 1 = mild (present but not disturbing sleep), 2 = moderate (awake once because of cough), 3 = severe (awake more than once because of cough), and 4 = extremely severe (insomnia throughout the night). Shortness of breath early in the morning: 0 = absent, 1 = mild (occasional and no medication required), 2 = moderate (occasional and medication required), 3 = severe (frequent and medication required), and 4 = extremely severe (persistent and multiple doses of medication required). Daytime wheezing or dyspnea: 0 = absent, 1 = mild (occasional wheezing), 2 = moderate (occasional wheezing and dyspnea but not disruptive of daily activities), 3 = severe (persistent and disruptive of daily activities), and 4 = extremely severe (completely prevents daily activities). Daytime cough: 0 = absent, 1 = mild (occasional but not disruptive of daily activities), 2 = moderate (frequent but not disruptive of daily activities), 3 = severe (frequent and disruptive of daily activities), and 4 = extremely severe (persistent) 26,28. Patients with total asthma symptom scores from 1 to 4, from 5 to 8, from 9 to 12, and from 13 to 16 were defined as having mild, moderate, severe, and extremely severe asthma symptoms, respectively 28.

### Subgroup classification

We divided the children into subgroups to evaluate the effect of stem cells on different groups. The children with asthma who had total IgE levels of <200 KU/L and >200 KU/L were categorized into lower (n = 15) and higher IgE (n = 30) asthma subgroups, respectively. The sex ratios in the lower and higher IgE asthma subgroups were 0.88:1 (7 girls and 8 boys) and 0.58:1 (11 girls and 19 boys), respectively. The average ages of children in the lower and higher IgE asthma subgroups were 5.79 years (5.79 ± 0.55) and 5.92 years (5.92 ± 0.41), respectively. No statistically significant intersubgroup age difference was noted (*P* = 0.84). The demographics of the participants are listed in Table 1.

### Molecular methods

#### PBMC isolation

We used density gradient centrifugation (Histopaque; Sigma Aldrich, St. Louis, MO, USA) to isolate PBMCs from heparinized venous blood. Initially, we depleted plasma through centrifugation (400 × *g* for 20 min). Residual blood cells were mixed with an equal volume of RPMI 1640 medium. Subsequently, Histopaque was added to the bottom of the centrifuge tube. The blood-RPMI 1640 mixture (the volume ratio of cells to Histopaque was 2:1) was slowly layered on the Histopaque solution. Next, the centrifuge tube was centrifuged at 400 × *g* at 20 °C for 30 min. By using a sterile pipette, we transferred the mononuclear cell layer to a second centrifuge tube. The cells were washed and collected after adding a 2-fold excess of Hanks' Balanced Salt Solution, and then the PBMCs were prepared for cell culture.

#### MSCs isolation

We isolated placenta-derived MSCs from the choriodecidual membrane of human placentas 29. The choriodecidual tissues donated by women who had undergone cesarean sections were digested using SMEM medium supplemented with 0.5 mg/mL protease, 0.5 mg/mL collagenase B, and 1 mg/mL DNase I at 4 °C overnight and filtered through a 100-μm nylon membrane. After centrifugation, cells were collected and subsequently resuspended in culture medium (MCDB201 supplemented with 1% insulin transferrin selenium, 10 ng/mL epidermal growth factor, and 1% penicillin/streptomycin). Next, the cells were plated in culture dishes coated with human collagen type IV. The adherent cells were kept in the culture medium; every 3 or 4 days, the medium was changed and the nonadherent cells were removed. Cells that were demonstrated to be placenta-derived MSCs were cultured in a serum-free medium, which displayed fibroblast-like morphology after attachment with a positive expression for CD29, CD44, CD73, CD90 and negative expression for CD14, CD34, CD45, and HLA-DR. *In vitro* assays of these cells showed positive signals for adipogenic, chondrogenic, and osteogenic differentiation when stained with Alcian Blue, Alizarin Red S, and Oil Red O, respectively 29.

### *In vitro* culture of PBMCs with or without MSCs

We cultured PBMCs at 2 × 10^5^ cells/mL in a RPMI 1640 medium in 96-well plates. These cells were stimulated with human anti-CD3 (0.5 μg/mL; Biolegend, San Diego, CA, USA) and human anti-CD28 antibodies (0.5 μg/mL; Biolegend) and cocultured with and without 10% placenta-derived MSCs (MSC:PBMC = 1:10). In the asthma group, we also used different concentrations of placenta-derived MSCs (MSC: PBMC = 1:5 and 1:20). After 72 hours; cell supernatants were harvested and frozen at -80 °C.

### Flow cytometry to detect cell activation and proliferation

The presence of the surface marker CD25 was detected in cultured cells to evaluate cell activation. These cells were stained with the fluorescent anti-CD25 antibody for 30 min at 4 °C and fixed in 1% paraformaldehyde. In addition, to evaluate cell proliferation, cells were stained with 5-µM carboxyfluorescein succinimidyl ester (CFSE) in phosphate-buffered saline (PBS) at room temperature for 5 min and then washed with 10 volumes of media with 10% PBS for 5 min before culturing. After culturing, all cells were examined for the presence of surface antigens by using flow cytometry.

### Detection of total IgE

We analyzed the total plasma IgE level by using an ImmunoCAP 250 analyzer (Phadia, Uppsala, Sweden). We added 40 µL of plasma to ImmunoCAP (Phadia) and incubated this mixture. The ImmunoCAP tube was then washed with a washing solution. Enzyme-labeled anti-IgE (conjugate against total IgE) was added to the ImmunoCAP and washed again. The bound total IgE was quantified using a fluorescent substrate. The ImmunoCAP testing system is highly automated (ImmunoCAP 250, Phadia, Uppsala, Sweden), and the process required 1.5 h in total.

### Enzyme-linked immunosorbent assay of the cytokine IL-5

We determined the IL-5 level by performing enzyme-linked immunosorbent assays with commercial human cytokine kits (R&D, Minneapolis, MN, USA). We measured the absorbance at 450 and 570 nm and subtracted the absorbance value obtained at 570 nm from that obtained at 450 nm to detect the IL-5 level. The absorbance was measured using the SpectraMax M5 multidetection microplate reader (Molecular Devices, Sunnyvale, CA, USA).

### Statistics

All data analyses were performed using STATA 12. Using the case-control method and paired *t*-tests, we evaluated the rates of activation and proliferation among CD4+ T cells and CD8+ T cells and compared them between the PBMC culture and PBMC + MSC (P+S) coculture in the asthma and nonasthma groups. We determined IgE and IL-5 levels and compared their values between the PBMC culture and P+S coculture in the asthma and nonasthma groups by using the case-control method and paired *t*-tests. The IL-5 levels in the subgroups were analyzed using one-way analysis of variance (ANOVA) followed by Bonferroni's analysis. A *P* value of <0.05 was considered significant.

## Results

### Analysis of total serum IgE

**Figure 1:**
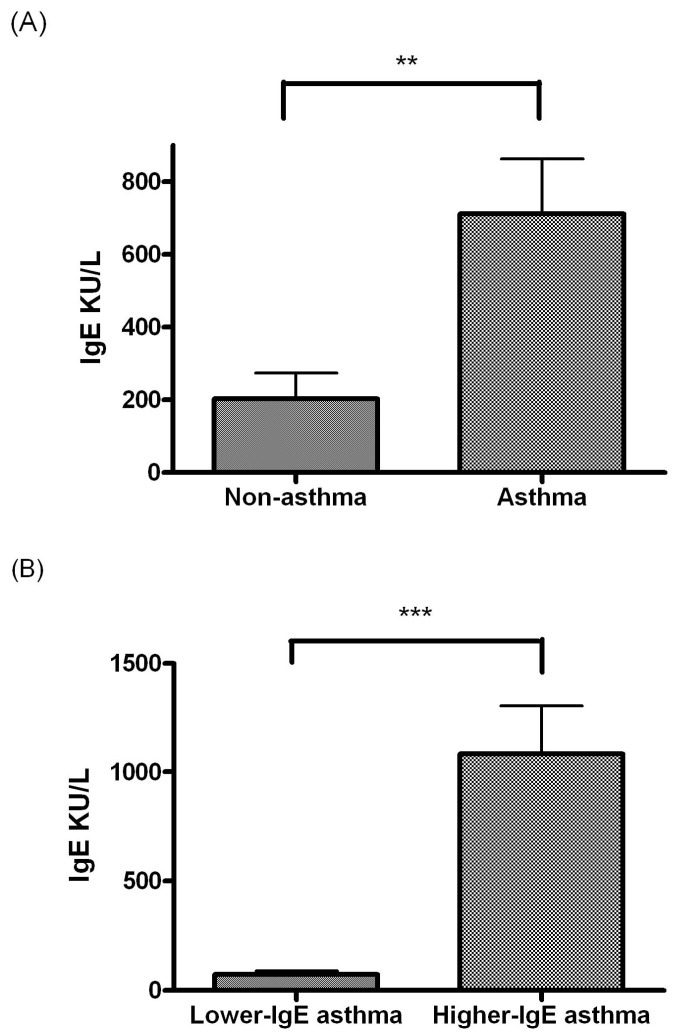
(A) Comparison of total IgE level between the children without asthma (n = 23) and children with asthma (n = 45). (B) Comparison of the total IgE level between lower and higher IgE asthma subgroups. * indicates *P* < 0.05; ** indicates *P* < 0.005; *** indicates *P* < 0.0005.

The total IgE level was significantly higher in the asthma group than in the nonasthma group (*P* < 0.005, Figure 1A). The total IgE level significantly differed between the lower and higher IgE asthma subgroups (*P* < 0.0005, Figure 1B).

### Analysis of activation and proliferation of CD4+ and CD8+ T cells in PBMC culture and P+S coculture

**Figure 2:**
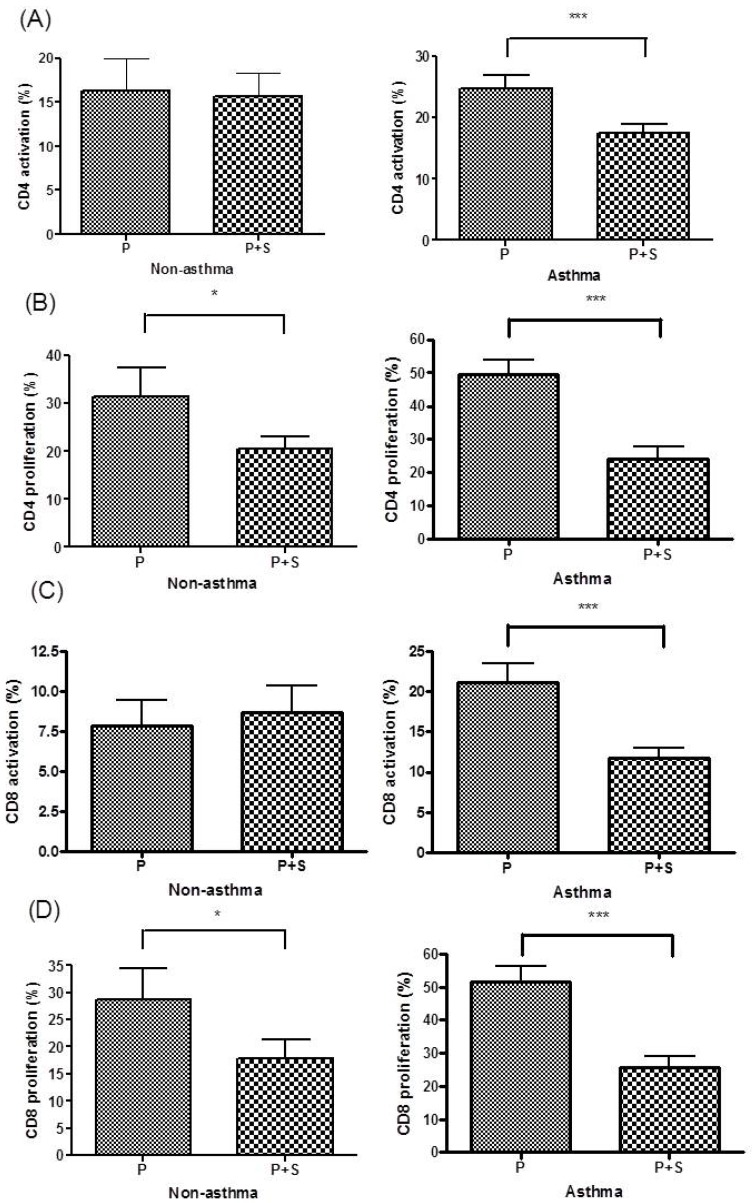
(A) Percentages of CD4+ T cell activation in the PBMCs (P) culture and PBMC + MSC (P+S) coculture in the nonasthma and asthma groups. (B) Percentages of CD4+ T cell proliferation in the P culture and P+S coculture in the nonasthma and asthma groups. (C) Percentages of CD8+ T cell activation in the P culture and P+S coculture in the nonasthma and asthma groups. (D) Percentages of CD8+ T cell proliferation in the P culture and P+S coculture in the nonasthma and asthma groups. * indicates *P* < 0.05; ** indicates *P* < 0.005; *** indicates *P* < 0.0005.

The rate of the activation of CD4+ T cells significantly differed between the PBMC culture and P+S coculture in the asthma group (*P* < 0.0005) but not in the children without asthma (Figure 2A). The rate of the proliferation of CD4+ T cells significantly differed between the PBMC culture and P+S coculture in both the asthma (*P* < 0.0005) and nonasthma groups (*P* < 0.05, Figure 2B). The rate of activation of CD8+ T cells significantly differed between the PBMC culture and P+S coculture in the asthma group (*P* < 0.0005) but not in the nonasthma group (Figure 2C). The rate of the proliferation of CD8+ T cells significantly differed between the PBMC culture and P+S coculture in both the asthma (*P* < 0.0005) and nonasthma groups (*P* < 0.05, Figure 2D).

### Analysis of the IL-5 level between the PBMC culture and P+S coculture

**Figure 3:**
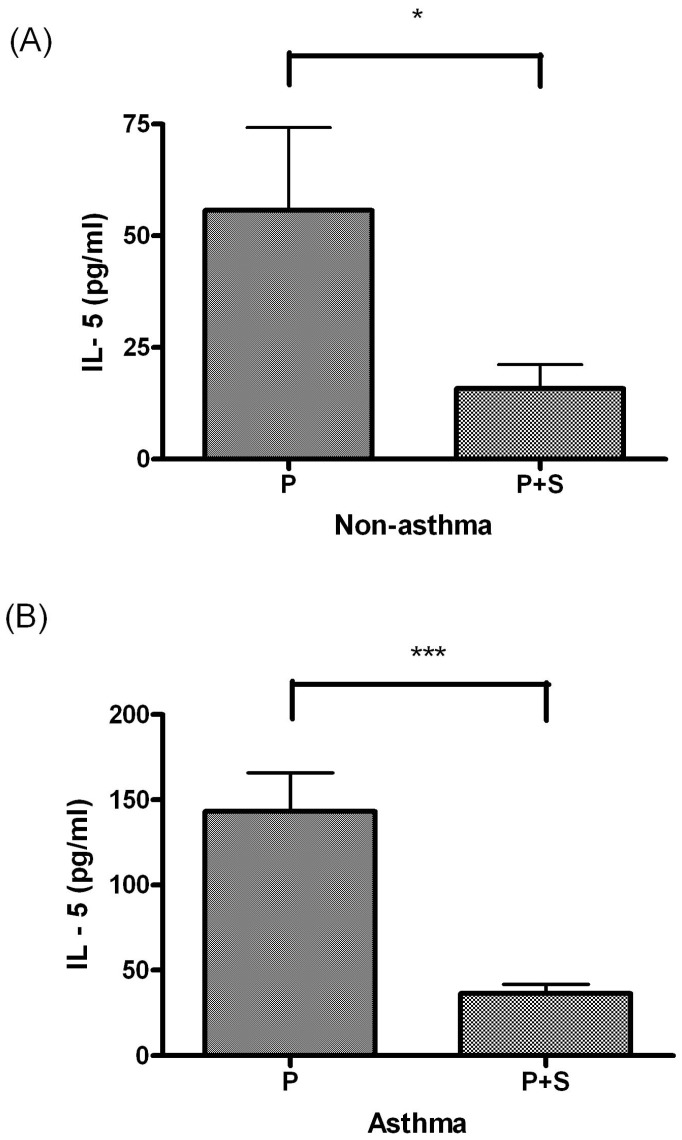
(A) Comparison of IL-5 levels between the PBMC (P) culture and PBMC + MSC (P+S) coculture in children without asthma. (B) Comparison of IL-5 levels between the P culture and P+S coculture in children with asthma. * indicates *P* < 0.05; ** indicates *P* < 0.005; *** indicates *P* < 0.0005.

IL-5 levels differed significantly between the PBMC culture and P+S coculture in the nonasthma (*P* < 0.05, Figure 3A) and asthma groups (*P* < 0.05, Figure 3B).

### Clinical severity in children with asthma

The asthma symptom scores of the asthma group, lower IgE asthma subgroup, and higher IgE asthma subgroup was 7.76 ± 0.56, 7.47 ± 0.8, and 7.9 ± 0.74, respectively. The asthma symptom score did not differ significantly between the lower and higher IgE asthma subgroups (*P* = 0.69, Table 1).

### Comparison of IL-5 levels between the PBMC culture and P+S coculture in the asthma subgroups

**Figure 4:**
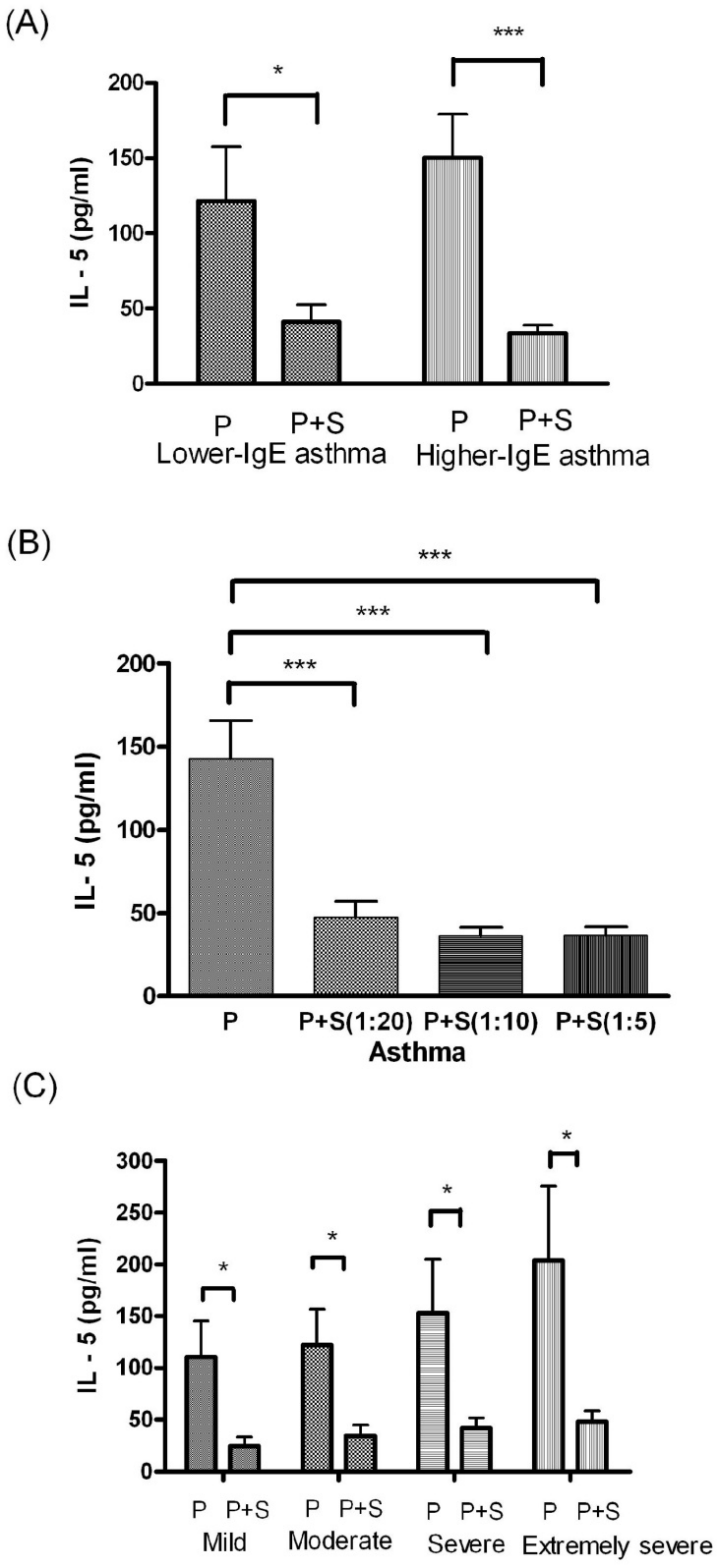
(A) Comparison of the IL-5 level between the PBMC (P) culture and PBMC+MSC (P+S) coculture in lower and higher IgE asthma subgroups. (B) Comparison of the IL-5 level between the P culture and P+S coculture in children with asthma. (C) Comparison of the IL-5 level between the P culture and P+S coculture in children with mild, moderate, severe, and extremely severe asthma symptom scores. * indicates *P* < 0.05; ** indicates *P* < 0.005; *** indicates *P* < 0.0005.

IL-5 levels significantly differed between the PBMC culture and P+S coculture in both the lower and higher IgE asthma subgroups (*P* < 0.05, Figure 4A). The results of one-way ANOVA revealed that IL-5 levels differed significantly between the PBMC culture and P+S coculture (different MSC concentrations) in the asthma group (F = 13.59, degrees of freedom = 142,* P* < 0.0005, Figure 4B). IL-5 levels differed significantly between the PBMC and P+S coculture and among the children with asthma who had mild (*P* < 0.05), moderate (*P* < 0.05), severe (*P* < 0.05), and extremely severe (*P* < 0.05) asthma symptom scores (Figure 4C).

## Discussion

Several studies have examined the mechanisms of MSCs in animal models, but none have focused on children with asthma. In murine models of asthma, MSCs were shown to be immunomodulatory and normalize the balance of type 1 T helper (Th1) cells and Th2 cells 19,21,23. The administration of MSCs in the murine models of asthma changes the phenotype of T lymphocytes from Th2 to Th1 cells 18,21. MSC therapy reduced airway eosinophilia in rodent models, regardless of the source of MSCs or the method of administration 17,18,19,22,24. Notably, the percentage of eosinophils was observed to decrease to its normal level several months after MSC treatment in cat models 25.

Murine studies have reported reductions in inflammatory cells and inflammatory mediators, such as tumor necrosis factor-α and IL-6, in both bronchoalveolar lavage fluid and serum after MSC transplantation 9. MSCs may also promote immune tolerance through the proliferation of Treg cells and the secretion of regulatory cytokines such as IL-10 in asthmatic mice 19,23. Transforming growth factor-beta (TGF-β) is a type of cytokine that causes airway inflammation and airway remodeling 30,31, and MSCs can suppress TGF-β by inhibiting alveolar macrophage polarization through the TGF-β signaling pathway 32. The overexpression of p-Akt was suppressed in the lung tissues of asthmatic rats after MSC transplantation 9. MSCs can prevent inflammatory infiltration around the airway deposition of collagen and airway remodeling in a rat model of chronic asthma by disrupting the PI3K/AKT signaling pathway 9. In a small number of adult patients with allergy-induced asthma, MSCs significantly reduced the interferon-γ level and increased the IL-10 level 33.

We postulated that in children with asthma, the mechanism of MSCs would involve the modulation of T cell immune responses and cytokine IL-5. In the present study, we evaluated the effects of MSCs on CD4+ T cells and CD8+ T cells by using an *in vitro* culture of PBMCs and a coculture of PBMCs with MSCs. Regarding T helper cells, we discovered that the activation of CD4+ T cells was significantly lower in the children with asthma treated with MSCs than in those not treated with MSCs. However, no significant differences were observed among the children without asthma between those who received and who did not receive MSC treatment. MSCs appeared to suppress the activation of T helper cells in PBMCs cultured *in vitro* after stimulation with antihuman CD3 and CD28 antibodies in the children with asthma but not in the children without asthma. In our study, we also found that CD4+ T cell proliferation was significantly lower in children with asthma who received MSC treatment than in those who did not receive MSC treatment. Significant differences were also noted in children without asthma. MSCs could suppress the proliferation of CD4+ T cells in PBMCs cultured *in vitro* after stimulation with antihuman CD3 and CD28 antibodies in both children with and without asthma. Regarding CD8+ T cells, we discovered that the rate of activation of CD8+ T cells was significantly lower in the children with asthma who received MSC treatment than in those who did not receive MSC treatment. However, no significant differences were noted between the children without asthma who received or did not receive MSC treatment. In addition, we observed that the proliferation of CD8+ T cells was significantly lower in children with asthma who received MSC treatment than in those who did not receive MSC treatment. Significant differences were also noted in children without asthma. MSCs could suppress the proliferation of CD8+ T cells in PBMCs cultured *in vitro* after stimulation with antihuman CD3 and CD28 antibodies in both the children with and without asthma. MSCs appeared to have a suppressive function that affected CD4+ and CD8+T cell activation and proliferation in children with asthma. Furthermore, we evaluated the effect of MSCs on IL-5 levels by using an *in vitro* culture of PBMCs cocultured with MSCs. IL-5 levels were significantly lower in the P+S coculture both in the children with and without asthma. MSCs appeared to exert an anti-IL-5 effect on the *in vitro* culture in both the children with and without asthma. Therefore, to evaluate the anti-IL-5 effect of MSCs in children with asthma under different conditions, we divided them into subgroups. We used different concentrations of MSCs for co-culturing with PBMCs. We observed that 5%, 10%, and 20% of MSCs cocultured with PBMCs exerted an anti-IL-5 effect. Notably, MSCs demonstrated a strong anti-IL-5 effect at different concentrations. In addition, we observed that the P+S coculture significantly reduced IL-5 levels in all of the children with asthma who had mild, moderate, severe, or extremely severe asthma symptom scores. This finding indicates that the MSCs exerted an anti-IL-5 effect in children with asthma with varying clinical severities. A clinical therapeutic study reported that the anti-IL-5 monoclonal antibody (mAb) inhibited eosinophil development and reduced the presentation of clinical asthma symptoms 34. Molfino et al. 35 reported that targeting IL-5 and its receptor is a rational therapy. The Food and Drug Administration of the United States has approved mepolizumab and reslizumab as new anti-IL-5 therapeutics for the treatment of patients with severe eosinophilic asthma 36. Mepolizumab is a type of humanized anti-IL-5 mAb that has been widely studied. Mepolizumab was found to improve FEV1 and reduce the number of eosinophils in the sputum and blood during asthma treatment 37.

Nonetheless, our study was performed on a small number of children and only focused on IL-5. Despite this limitation, the results revealed significant differences after MSC coculture. We will conduct further studies in the future to evaluate a range of Th2-associated cytokines (e.g., IL-4 and IL-13) and also test current concepts regarding the addition of MSCs with different T cell subsets, such as Th9 (e.g., IL-9), and regulatory T cells (e.g., IL-10, TGFβ, and FoxP3). Our study observed that placenta-derived MSCs undoubtedly exerted an anti-IL-5 effect in culture, and can therefore be considered a potential treatment for asthma.

In summary, our study results demonstrated that placenta-derived MSCs could suppress the proliferation of CD4+ T cells and CD8+ T cells in all children and suppress the activation of CD4+ T cells and CD8+ T cells in children with asthma. Placenta-derived MSCs exerted an anti-IL-5 effect and reduced IL-5 levels in an *in vitro* culture in different subgroups of the children with asthma. Notably, in the culture, MSCs at different concentrations exhibited the ability to suppress IL-5 in children with asthma. According to our study results, placenta-derived MSCs can be considered a potential cell-based therapy for asthma. Therefore, placenta-derived MSCs may be used in children with asthma who respond poorly to conventional treatments.

## Figures and Tables

**Table 1:** Participant Demographics

Number of children (total)	68
Ratio of children without asthma to those with asthma	23:45
Ratio of lower to higher IgE asthma	15:30
Ratio of girls to boys (total)	28:40
Nonasthma (girls:boys)	10:13
Asthma (girls:boys)	18:27
Lower IgE asthma (girls:boys)	7:8
Higher IgE asthma (girls:boys)	11:19
Mean age (total)	6.00 ± 0.26
Age ratio of nonasthma to asthma	6.25 ± 0.44:5.88 ± 0.32
Age ratio of lower to higher IgE asthma	5.79 ± 0.55:5.92 ± 0.41
Mean asthma symptom score (asthma) Asthma symptom score ratio of lower to higher IgE asthma	7.76 ± 0.56 7.47 ± 0.8:7.9± 0.74

## Abbreviations

Th2: type 2 T helper cell
Th1: type 1 T helper cell
IL: interleukin
MSCs: mesenchymal stem cells
iPSC-MSCs: induced pluripotent stem cell-derived MSCs
Treg: regulatory T
PBMCs: peripheral blood mononuclear cells
PBS: phosphate-buffered saline
ANOVA: one-way analysis of variance
TGF-β: transforming growth factor-beta
